# Common mental disorders amongst frontline healthcare workers during the COVID-19 pandemic in Ethiopia: A cross-sectional study

**DOI:** 10.4102/sajpsychiatry.v28i0.1733

**Published:** 2022-02-24

**Authors:** Mandaras Tariku, Tilahun Ali, Tadesse Misgana, Dejene Tesfaye, Daniel Alemu, Yadeta Dessie

**Affiliations:** 1 Department of Psychiatry, College of Health and Medical Sciences, Haramaya University, Harar, Ethiopia; 2 School of Public Health, College of Health and Medical Sciences, Haramaya University, Harar, Ethiopia

**Keywords:** COVID-19, Ethiopia, frontline, health workers, mental health problems, SARS-CoV-2

## Abstract

**Background:**

A novel coronavirus had a profound physiological and psychological burden with regards to contracting the disease or uncertainties in the care of infected patients. Especially, at risk are frontline healthcare workers who are participating in the care of such patients.

**Aim:**

This study investigated the burden of mental health problems amongst the frontline health workers during the coronavirus disease 2019 (COVID-19) pandemic in Ethiopia.

**Setting:**

East Hararghe Zone of Oromia Region and Harari Regional State, Ethiopia.

**Methods:**

A cross-sectional study was conducted in three selected hospitals of COVID-19 treatment centers. Simple random sampling was used to select a sample of 423 participants from each hospital. The self-Reporting Questionnaire (SRQ-20) was used to assess the presence of common mental disorders. Binary and multivariable logistic regressions were fitted to identify factors associated with common mental disorders. Statistical significance was declared at a p-value less than 0.05.

**Results:**

The prevalence of common mental disorders amongst frontline healthcare workers was 22.6%. Being female, married, having had direct contact with COVID-19 patients, working in COVID-19 treatment centers and ICU, having any symptoms of COVID-19, current three-month use of any substances, and poor social support were found to be strong predictors of common mental disorders in frontline health workers during COVID-19 pandemic in Ethiopia.

**Conclusion:**

The considerable proportions of frontline health care workers have common mental health problems. Strategies need to address COVID-19 related mental health problems, and integrate psychosocial intervention to support the frontline health workers is paramount.

## Introduction

Coronavirus disease 2019 (COVID-19) is complex in mode of transmission, which has high impact on the economic growth, increased demand of healthcare facilities and impact on social aspects which could affect the world population at large.^1,2^ It has an enormous impact on the health system and primarily the frontline workers.^3,4^ Around 3.8% of medical workers have been infected, mainly because of early unprotected contact with infected patients.^4,5^

As the pandemic rapidly spreads across the world, it is inducing a considerable degree of fear, worry and concern in the population at large and amongst certain groups in particular.^1,6^ Healthcare providers, the elderly, children, including individuals with underlying health conditions were identified as at-risk.^7^ Healthcare workers, because of the nature of their work are at the forefront in the fight against the virus compared to the rest of the population.^8^ Thus, maintaining an adequate healthcare workforce in this crisis requires not only an adequate number of healthcare providers but also maximising the efficiency through psychosocial supports (supportive communication in everyday interaction and supporting the healthcare workers who are experiencing distress), and addressing issues which could hinder them from managing patients with COVID-19.^8,9^

The primary exposed groups during the time of an infectious disease outbreak are the frontline healthcare workers.^10^ As the health system became inundated with patients, the resources needed to protect healthcare workers from infection became increasingly scarce and in developing countries where the facility of health delivering systems is not well organised, front-line workers for COVID-19 are more stressed and prone to the development of different categories of mental health problems such as anxiety, suicide, sleep problems, depressive disorders and became martyrs.^10,11,12^ Early identification and intervention of psychological problems associated with COVID-19 are pivotal to ensure an appropriate response to this pandemic.^13,14^

The common mental disorders (CMD) are high during this pandemic in frontline healthcare workers; these could be because of the burden of their setting, wearing heavy protective personal equipment, making it much more difficult to carry out medical operations or procedures than under normal conditions.^15^ In addition, strict procedures to be followed, higher demands in the work setting, reduced capacity to use social support and stigma within the communities are putting them at further risk.^16,17^ These factors, together with the fear of being contagious and infecting others (including the loved ones), could increase the possibility of developing CMD amongst frontline workers.^18,19^

Therefore, it is very important to study frontline health workers’ mental health status and the burden of psychological disorder with regard to COVID-19 preparedness or care provision. There are several studies conducted in the developed countries that showed the mental health problems of frontline healthcare workers, but there are limited findings in developing countries. Although the importance of mental wellness amongst healthcare workers is well understood, the burden of common mental health problems related to COVID-19 is not well studied yet in Ethiopia.

## Methods

### Study design

A facility-based cross-sectional study was conducted in three selected hospitals of COVID-19 treatment centres in Eastern Ethiopia.

### Setting

The hospitals (Haramaya Hospital, Hiwot Fana Specialised University Hospital and Turkish temporary COVID-19 treatment centre) were found in East Hararghe Zone of Oromia Region and Harari Regional State, Eastern Ethiopia. These hospitals were prepared to provide service to patients with COVID-19.

### Study population

All frontline healthcare workers involved in COVID-19 management in East Hararghe Zone of Oromia Region and Harari Regional State, Eastern Ethiopia were the source of population and the healthcare workers who were available during the data collection period were the study population for this study. Participants who were invited, but failed to respond to three calls and an SMS reminder at different times were excluded from the study.

### Sample size, sampling technique and procedure

The sample size determination was estimated using a single population proportion formula (Equation 1):


$$n=\frac{{\left(z_{\alpha /2}\right)}^{2}p(1-p)}{d^{2}}$$ [Eqn 1]

Considering 95% confidence interval (CI), 50% population proportion with a 5% margin of error, the minimum sample size was 384. Including 10% of the non-response rate, the final sample size was 423. The samples were collected from each hospital using simple random sampling. The sampling frame was taken from the COVID-19 task forces of the region. All COVID-19 protective measures were kept during the data collection period.

### Data collection tool and procedure

The data were collected through self-administered questionnaires from randomly selected study participants. A semi-structured questionnaire was used to collect socio-demographic, COVID-19 and clinical-related variables.

Oslo social support scale and Alcohol, Smoking and Substance Involvement Screening Test (ASSIST) were used to assess the social support and substance characteristics of the participants. Based on Oslo social support scale, the score (3–8) was poor social support, (9–11) was moderate social support and (12–14) was strong social support.^20,21^ Based on ASSIST, current substance use was by those who used (non-medical use only) at least one substance for the last 3 months.^22^ A Self-Report Questionnaire (SRQ-20) was used to screen the presence of CMD.^23^ The tool is composed of 20 items rated to 1 ‘Yes’ and 2 ‘No’. It contains the experience of depressive, anxiety and somatic symptoms in the preceding 30 days. The cut-off point > 7 was considered the presence of CMD.^24^

### Data management and analysis

Data were checked for completeness and consistency. It was coded and entered using EpiData version 3.1 and analysed by using statistical package software for social sciences (SPSS) version 20. Binary and multivariable logistic regression was used to identify the factors associated with CMD. Adjusted odds ratio (AOR) with 95% CI and *p*-value less than 0.05 was considered statistically significant.

### Ethical considerations

Ethical clearance was obtained from Haramaya University College of Health and Medical Science Institutional Health Research Ethics Review Committee (IHRERC). The letter of permission was written to the respective regional administrator and COVID-19 task forces of the region. The purpose and the risk and benefit of the study were explained to the participants. The participants were informed that participation in the study is voluntary and have the right to withdraw or refuse to participate in the study at any time. During the data collection, all WHO recommendations for the prevention of COVID-19 infection were followed.

## Results

### Socio-demographic description of the study participants

The study participants’ response rate was 95.03% (402/423). The mean age was 29.4 (± 5.7) years. A total of 169 (42%) study participants were female. A considerable portion of 137 (34.1%) participants were married. Considering the profession of the participants, 165 (41.0%) were nurses, and 95 (23.6%) were medical doctors. About the educational status of the study participants, the majority 317 (78.9%) of the frontline healthcare workers had a degree and above (Table 1).

**TABLE 1:** Socio-demographic description of frontline healthcare workers during COVID-19 pandemic in Ethiopia, 2021 (*n* = 402).

Variables	Category	Frequency	Percentage
Gender	Male	233	58.0
	Female	169	42.0
Marital status	Single	170	42.3
	Married	137	34.1
	Others†	95	23.6
Ethnicity	Oromo	221	55.0
	Amhara	113	28.1
	Harari	37	9.2
	Others‡	31	7.7
Educational status	Diploma	85	21.1
	Degree and above	317	78.9
Profession	Nurse	165	41.0
	Medical doctor	95	23.6
	Pharmacy	74	18.4
	Laboratory	19	4.7
	Health officer	26	6.5
	Others§	23	5.7

† , Divorced, widowed and separated.

‡ , Gurage, Tigrai, Silte.

§ , Anesthetics, ophthalmologist.

### Clinical and COVID-19 related variables of the study participants

As Table 2 shows, the mean year of experience for the study participants was 5.6 (± 4.8) years during the data collection period. More than half of the 226 (56.2%) respondents have had direct contact with COVID-19 patients during their working area. Regarding the professionals’ current working unit, 86 (21.4%) were working in COVID-19 treatment centres, and 36 (9%) were working in intensive care units (ICUs). It was also noted that 117 (29.1%) professionals were working in the ward to deliver healthcare services for the patients. As to the time focusing on gathering information about the current pandemic, more than half of the 254 (63.2%) respondents spent two times daily on focusing information about COVID-19. Regarding COVID-19 symptoms of the study participants, 93 (23.1%) have had COVID-19 symptoms during the data collection period (Table 2).

**TABLE 2:** Clinical and COVID-19 related description of the frontline healthcare workers in Ethiopia, 2021 (*n* = 402).

Variables	Category	Frequency	Percentage
Year of experience	Mean (s.d.)	5.6	4.8
Contact with COVID-19 patients	Yes	226	56.2
	No	176	43.8
Working unit	COVID-19 treatment centre	86	21.4
	ICU	36	9.0
	OPD	94	23.4
	Ward	117	29.1
	Others	69	17.2
Adequate personal protective equipment	Yes	234	58.2
	No	168	41.8
Time spent focusing on gathering COVID-19 information	None	31	7.7
	Two times daily	254	63.2
	3 and more times	117	29.1
Showed some COVID-19 symptoms	Yes	93	23.1
	No	309	76.9

s.d., standard deviation; COVID-19, coronavirus disease 2019; ICU, intensive care unit; OPD, outpatient department.

**FIGURE 1:**
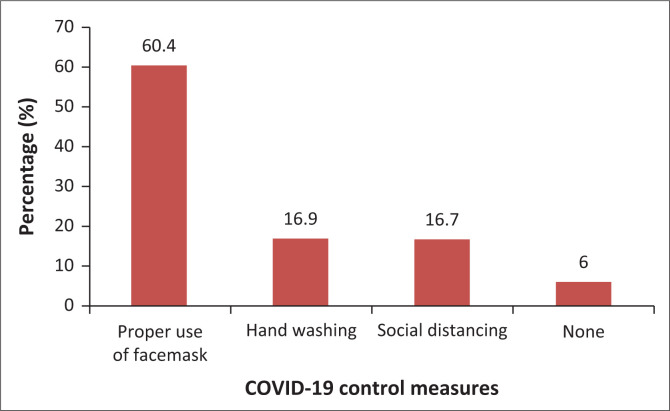
COVID-19 control measures mostly practised by the frontline healthcare workers in Ethiopia, 2021.

The frontline healthcare workers have had to practise different measures of COVID-19 during the time of data collection. Accordingly, 243 (60.4%), 68 (16.9%) and 67 (16.7%) of them were mostly practising proper use of mask, hand washing and social distancing, respectively, but 25 (6%) of them were not practising any type of COVID-19 control measures during the study period (Figure 1).

### Social support description of the study participants

The social support level of the frontline healthcare workers was assessed by using Oslo social support scale which was grouped into poor, moderate and strong social support. Accordingly, 181 (45%) of them had poor social support, 154 (38.3%) had moderate social support and 67 (16.7%) had strong social support.

### Substance use description of the study participants

Concerning any substance use in the lifetime and current time, majority of the frontline healthcare workers 290 (72.1%) never used any substance in their lifetime, and 96 (23.9%) study participants were using some type of substance in the current 3 months.

### Common mental disorders amongst frontline healthcare workers during COVID-19 pandemic in Ethiopia

**FIGURE 2:**
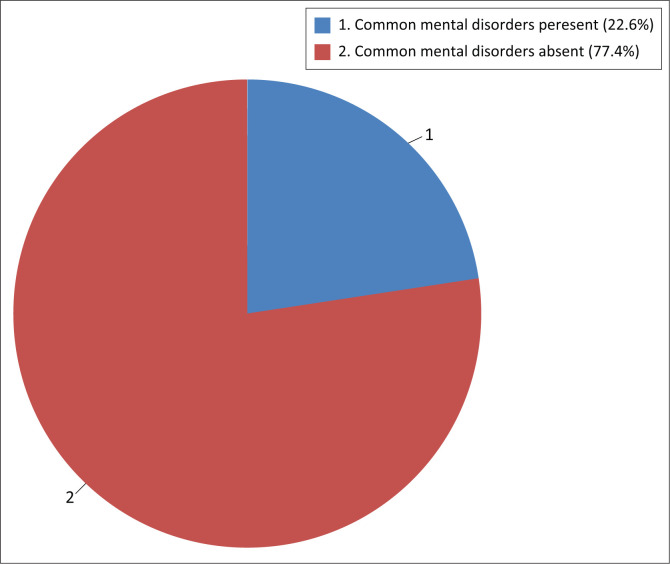
Prevalence of common mental disorder amongst frontline healthcare workers amidst COVID-19 pandemic in Ethiopia, 2021.

Common mental disorders were measured using SRQ-20 and a score more than 7 (seven) denoted the presence of CMD. Based on this finding, the prevalence of the CMD amongst frontline healthcare workers was 91 (22.6%) with 95% CI (18.4, 26.4) (Figure 2).

### Factors associated with common mental disorders amongst frontline healthcare workers

In this study, sex, marital status, having direct contact with COVID-19 patients, working units, having any symptoms of COVID-19, current 3-month use of any substances and poor social support were found to be strong predictors of CMD amongst frontline healthcare workers in East Ethiopia (Table 3).

**TABLE 3:** Factors associated with common mental disorders amongst frontline healthcare workers during COVID-19 pandemic in Ethiopia, 2021 (*n* = 402).

Explanatory variable	Category	Common mental disorder		COR, 95% CI	AOR, 95% CI
		Yes	No		
Gender	Female	61	108	3.82 (2.33, 6.30)	4.50 (2.42, 8.43)**
	Male	30	203	1	1
Marital status	Married	45	92	2.60 (1.50, 4.50)	3.89 (1.90, 7.95)**
	Others	19	76	1.32 (0.70, 2.54)	1.51 (0.66, 3.50)
	Single	27	143	1	1
Direct contact with COVID-19 patients	Yes	69	157	3.10 (1.81, 5.22)	2.80 (1.45, 5.40)*
	No	22	154	1	1
Working unit	COVID-19 treatment centre	32	43	4.00 (2.00,7.84)	3.45 (1.45, 8.20)*
	ICU	17	26	3.49 (1.58, 7.70)	2.15 (0.79, 5.85)*
	OPD	12	89	0.72 (0.33, 1.60)	0.71 (0.30, 1.80)
	Others	12	57	1.12 (0.50, 2.50)	0.94 (0.35, 2.51)
	Ward	18	96	1	1
Showed some symptoms of COVID-19	Yes	42	49	4.40 (2.62, 7.30)	4.00 (2.10, 7.64)**
	No	51	260	1	1
Presence of adequate PPE	Yes	60	31	1.52 (0.94, 2.50)	0.85 (0.44, 1.62)
	No	174	137	1	1
Current use of any substance	Yes	29	62	1.70 (1.02,2.86)	2.62 (1.30, 5.40)*
	No	67	244	1	1
Educational background	Diploma	24	61	1.50 (0.85. 2.50)	1.55 (0.73, 3.30)
	Degree and above	67	250	1	1
Years of experience	-	-	-	0.92 (0.86, 0.98)	0.90 (0.82, 0.96)*
Social support	Poor	60	121	2.06 (1.04, 4.06)	2.52 (1.01, 6.30)*
	Moderate	18	136	0.55 (0.25, 1.20)	0.50 (0.20, 1.33)
	Strong	13	54	1	1

COR, Crude odds ratio; AOR, adjusted odds ratio; CI, confidence interval; COVID-19, coronavirus disease 2019; ICU, intensive care unit; OPD, outpatient department.

** , *p* < 0.001;

* , *p* < 0.05: Significant association; *df* = 8, Hosmer and Lemeshow = 0.68.

About the sex of the frontline healthcare workers, female participants were found to be 4.5 times more likely to develop CMD (AOR = 4.50, 95% CI = 2.42, 8.43) (Table 3).

The frontline healthcare workers who were married were found to be 3.89 times more likely to develop CMD compared with single frontline healthcare workers during the COVID-19 period (AOR = 3.89, 95% CI = 1.90, 7.95) (Table 3).

The participants who had direct contact with COVID-19 patients were found to be 2.80 times more likely to develop CMD compared with the participants who had no direct contact with COVID-19 patients (AOR = 2.80, 95% CI = 1.45, 5.40) (Table 3).

Regarding the frontline healthcare workers working unit, the study participants who had worked in COVID-19 treatment centres and ICUs were found to be 3.45 and 2.15 times more likely to have CMD, respectively, compared with the study participants who were working in the ward (AOR = 3.45, 95% CI = 1.45, 8.20 and AOR = 2.15, 95% CI = 0.79, 5.85) (Table 3).

About the frontline healthcare workers who had some symptoms of COVID-19, the participants who had some symptoms of COVID-19 were found to be 4.00 times more likely to develop CMD compared with the study participants who did not experience sign and symptoms of COVID-19 (AOR = 4.00, 95% CI = 2.10, 7.64) (Table 3).

The study participants who had used any type of substance in the last 3 months, and those with poor social supports were 2.62 and 2.52 times more likely to have had developed CMD, respectively (AOR = 2.62, 95% CI = 1.30, 5.40 and AOR = 2.52, 95% CI = 1.01, 6.30) (Table 3).

## Discussion

This study determined the prevalence and factors associated with CMD amongst frontline healthcare workers during the time of the COVID-19 global pandemic. Accordingly, the prevalence of the CMD amongst frontline healthcare workers who were fighting COVID-19 disease in Ethiopia was 22.6% (18.4–26.4). The finding is low compared with the results from China which revealed that a considerable proportion of participants reported symptoms of depression (50.4%), anxiety (44.6%), insomnia (34.0%) and distress (71.5%).^7^ The lower proportion of the current finding might be because of the time of the first exposure of frontline healthcare workers in the absence of adequate personal protective equipment and lack of enough information about the transmission, clinical manifestation and the course of novel coronavirus. This finding is also lower compared with the study conducted during the SARS outbreak in China which showed that 68% of the frontline healthcare workers experienced a high level of stress and 57% developed psychological distress. The lower finding of the current study might be because of the adaptability of the healthcare workers with the COVID-19 pandemic and learned experience from other countries affected by COVID-19.

This study also showed that being female sex, married, having a direct contact with COVID-19 patients, working in COVID-19 treatment centres and ICUs, having any symptoms of coronavirus, current 3-month use of any substances and poor social support were found to be strong predictors of CMD amongst frontline healthcare workers.

The odds of developing the common mental health problems during the COVID-19 pandemic amongst female and married frontline healthcare workers were 4.5 and 3.89 times high, respectively. There were consistent findings from a study conducted in Iran^25^ concluding that the prevalence of anxiety was high amongst female participants. This finding was an inconsistent result with a previously conducted study in Singapore^26^ which stated that single healthcare workers (AOR = 1.4, 95% CI = 1.02, 2.0) developed more psychiatric symptoms during the SARS outbreak. This inconsistent result might be because of an adequate sample of the study participants, psychosocial stressors underlying the occurrence of mental health problems, and the distribution of study participants was different from the current study. The high prevalence of CMD amongst married frontline healthcare workers might be because of the fear of being infected and transmitted to their family members including their child or loved one, and the stress of running a household.^27^

The prevalence of CMD was 2.80 and 3.45 times high amongst healthcare workers who had direct contact with patients of coronavirus and had some symptoms of COVID-19, respectively. This result is supported by a previously conducted study in China.^28^ The high prevalence of mental health problems of those who had direct contact experiencing some symptoms of COVID-19 might be the fear of the lethal effect of coronavirus, fear of the risk of transmission of disease to their families, peer or loved ones.^29^

The healthcare workers who were working in COVID-19 treatment centres and ICUs were found to be 3.45 and 2.15 times more likely to have CMD, respectively, compared with the study participants who were working in the ward. The result is consistent with the finding from China^14^ that showed that the medical staff working in the COVID-19 treatment centres had a higher prevalence of common mental health problems.

This study also revealed the current use of any type of substance and poor social support as predictors of mental health problems during the COVID-19 pandemic. There were observed the high prevalence of CMD amongst participants who had used any substance in the current 3 month and poor social support. The finding supports that good social support is a vital factor in protecting the healthcare workers’ mental well-being and psychological health.^6,30,31^

Furthermore, the finding from this study recognises that the long-term working experience in the hospital was protective for the occurrence of CMD. As the work experience is increased by a year, the development of common mental health problems is decreased by 0.9. The low occurrence of mental disorders in the long-duration of work might be because of the life experience gained by the healthcare workers, good copy strategies and interaction with the peer group.

## Limitation of study

Because of its cross-sectional nature, the study could not explore the cause and effect relationship between the outcome and predictors. In the current study, the presence of major stressful life events and the role of the participants in the families were not assessed: this could be the potential to the confounding variables for this study.

## Conclusion

Considerable proportions of frontline healthcare workers have common mental health problems. Being female, married, having had direct contact with COVID-19 patients, working in COVID-19 treatment centres and ICUs, having some symptoms of COVID-19, current 3-month use of any substance and poor social support were significantly associated with CMD. Therefore, strategies need to address problems related to mental health and integrate psychosocial intervention to support the frontline healthcare workers.

Establishing psychosocial support such as supportive communication in everyday interaction and supporting the healthcare workers who are experiencing distress are of significance to reduce the impact of COVID-19 amongst frontline healthcare workers. The female, married and frontline healthcare workers who have poor social support pay substantial attention to control the disastrous mental health effect of the coronavirus pandemic amongst healthcare workers.
